# Structure Characterization and Otoprotective Effects of a New Endophytic Exopolysaccharide from Saffron

**DOI:** 10.3390/molecules24040749

**Published:** 2019-02-19

**Authors:** Juan Li, Guimei Wu, Cuiying Qin, Wuhai Chen, Gang Chen, Lu Wen

**Affiliations:** 1 School of Pharmacy, Guangdong Pharmaceutical University, Guangzhou 510006, China; smilewinnie@126.com (J.L.); gdpuwgm@126.com (G.W.); gdpuwen@126.com (C.Q.); gdpucwh@126.com (W.C.); 2 Guangdong Provincial Key Laboratory of Advanced Drug Delivery, Guangdong Pharmaceutical University, Guangzhou 510006, China; 3 Guangdong Provincial Engineering Center of Topical Precise Drug Delivery System, Guangdong Pharmaceutical University, Guangzhou 510006, China

**Keywords:** hearing loss, saffron, endophytic exopolysaccharide, gentamicin, cochlear hair cell

## Abstract

Saffron, a kind of rare medicinal herb with antioxidant, antitumor, and anti-inflammatory activities, is the dry stigma of *Crocus sativus* L. A new water-soluble endophytic exopolysaccharide (EPS-2) was isolated from saffron by anion exchange chromatography and gel filtration. The chemical structure was characterized by FT-IR, GC-MS, and 1D and 2D-NMR spectra, indicating that EPS-2 has a main backbone of (1→2)-linked α-d-Manp, (1→2, 4)-linked α-d-Manp, (1→4)-linked α-d-Xylp, (1→2, 3, 5)-linked β-d-Araf, (1→6)- linked α-d-Glcp with α-d-Glcp-(1→ and α-d-Galp-(1→ as sidegroups. Furthermore, EPS-2 significantly attenuated gentamicin-induced cell damage in cultured HEI-OC1 cells and increased cell survival in zebrafish model. The results suggested that EPS-2 could protect cochlear hair cells from ototoxicity exposure. This study could provide new insights for studies on the pharmacological mechanisms of endophytic exopolysaccharides from saffron as otoprotective agents

## 1. Introduction

Hearing loss is a global problem. To date, more than 466 million people have moderate to severe or greater hearing loss, and one-third of them are over 65 years old. As the world’s population ages, it is estimated that approximately 900 million people (or one in every 10 people) will suffer hearing loss by 2050. World Health Organization (WHO) estimates that an annual global cost of hearing loss will be US $750 billion, including health sector costs (excluding hearing equipment), education support costs, productivity losses and social costs [1]. Unfortunately, hearing loss has not received sufficient attention by the pharmaceutical industry, and until now there have been few Food and Drug Administration (FDA)-approved drugs to treat or prevent different types of hearing loss [2]. Most hearing loss is caused by permanent loss of hair cells in the inner ear. One of the most likely causes of hair cell death is exposure to ototoxic agents, including aminoglycoside antibiotics such as gentamicin (GM) and neomycin, and cisplatin anticancer agents [3,4]. Additionally, it is estimated that aminoglycoside antibiotics generate hearing thresholds of almost 50% [5]. However, these aminoglycoside drugs also continue to be used in view of their cost and effectiveness, and their ototoxicity usually limits the dose range of drugs [4,6]. GM is a cationic aminoglycoside that enters cells via endocytosis and forms a complex with iron, which drives the formation of free radicals and directly promotes the formation of ROS [7]. Recent reports show that antioxidant drugs can benefit patients with hearing loss, because hearing loss is produced by excessive generation of reactive oxygen species (ROS) in cells of the cochlea [8,9]. Therefore, it is highly possible to find potential drugs in antioxidants to prevent hair cell loss due to ototoxicity exposure and attenuate hearing loss.

With today’s interest in new renewable sources of polymers, polysaccharides are macromolecular carbohydrates that play a vital role in the growth and development of living organisms, and serve as important biological response modifiers. Furthermore, it is well known that the polysaccharides can be used in various pharmaceutical formulations due to their sustainability, biodegradability, and biosafety [10,11,12]. Polysaccharides are widely distributed in plants, animals, and microorganisms. Endophytic fungi symbiotically live in plant tissues and all or part of their entire life cycles is spent in and/or between plant cells, often without causing apparent symptoms of diseases. Moreover, these microorganisms play important roles as components of plant ecosystems [13]. Endophytic fungi benefit their hosts by enhancing resistance to disease, abiotic stress and plant growth, and they have been widely recognized as a rich, potential and novel source of natural bioactive substances in agricultural, pharmaceutical and food industries [14]. Nowadays, endophytic fungi, as a renewable resource, are of growing interest. They often produce exopolysaccharides with unique structures and diverse biological activities, which have become the most promising group of antioxidants [15,16].

Saffron (*Crocus sativus* L.) is a perennial stemless herb of the Iridaceae family grown in Turkey, Iran, and China, and it has a variety of biological activities, such as antitumor, antioxidant, and anti-inflammatory effects [17,18]. As the main medicinal part of saffron, the stigma extract has antioxidant properties, which can scavenge oxygen radicals and effectively inhibit the activity of oxygen radicals [19]. Our previous studies have shown that crude exopolysaccharide extracted from fermentation mycelia of saffron exhibited excellent scavenging activities against 1, 1-diphenyl-2-picrylhydrazyl, hydroxyl and superoxide anion radicals [20]. However, the purification of exopolysaccharide and its further activity have not been researched. The present research describes the purification and characterization of a water-soluble exopolysaccharide (EPS-2) from the fermentation culture of endophytic fungus *Penicillium citreonigrum* CSL-27 of saffron and investigates its protective role in the damaged cochlear hair cells.

## 2. Results and Discussion

### 2.1. Isolation and Purification of Exopolysaccharide

**Figure 1:**
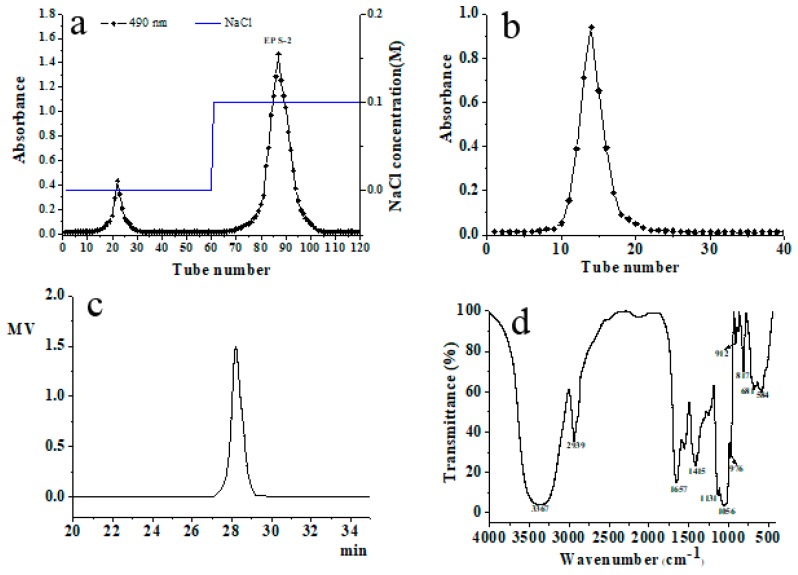
Chromatograms of EPS-2 from *P. citreonigrum* CSL-27 from (**a**) DEAE-52 cellulose column chromatography, (**b**) Sephadex G-75 chromatography, (**c**) HPGPC, and (**d**) FT-IR spectrum.

The main exopolysaccharide fraction EPS-2 was collected according to the detection curve of phenol-sulphuric acid colorimetry. After purification with a DEAE-52 cellulose column (Figure 1a), one major fractional peak was obtained. According to Figure 1b, the fraction appeared as a single and symmetrical peak after being further purified by Sephadex G-75 column chromatography. EPS-2 appeared as a single and symmetrical peak in the high performance gel permeation chromatography (HPGPC) (Figure 1c), indicating homogeneity. By comparison of the retention times of EPS-2 with the molecular standards, the molecular weight of EPS-2 was estimated to be 40.4 kDa. The colorimetric analysis has shown that EPS-2 contains 88.9% total carbohydrate, and no sulfate ester, protein, or uronic acid is detected. Monosaccharide composition analysis indicated that EPS-2 was mainly composed of mannose, glucose, galactose, xylose, and arabinose with a molar ratio of 51.77:36.76:5.76:3.16:6.94. The results of SEM have shown that EPS-2 has a frizzy shape, and the surface has a scaly texture. The irregular aggregation has determined that EPS-2 is an amorphous solid. The molecular aggregation may be attributed to a repulsive force between the polysaccharides and the side chains.

### 2.2. Fourier Transformed Infrared (FT-IR) Spectroscopy Analysis

The IR spectrum of EPS-2 displayed the characteristic peaks of polysaccharide (Figure 1d). The strong and broad absorption peak at 3367 cm^−1^ was related to the stretch vibration of O–H (hydroxyl group) bond existing in all polymers. The strong peak at 2939 cm^−1^ was due to the C–H stretching vibration in the sugar ring and the strong absorption peak at 1657 cm^−1^ represented the stretching vibration of C=O and carboxyl group. Another peak at 1418 cm^−1^ could be attributed to the symmetric stretching of the COO-group [21]. The presence of strong absorbance in the region 1200–950 cm^−1^ indicated the polysaccharide nature of EPS-2. The strong absorption at 1131 and 1056 cm^−1^ in the range of 1200–1000 cm^−1^, which is anomeric region, was attributed to C–O–C and C–O groups in the polysaccharide, suggesting that the monosaccharide in the EPS-2 has a pyranose ring [22]. Moreover, the band at 912 cm^−1^ indicated the pyranose form of the glucosyl residue and absorption peak at 817 cm^−1^ as well as the existence of glycosidic linkages of the EPS-2. Moreover, the weak absorption at 912 and 817 cm^−1^ was assigned to the coexistence of α and β glycosidic bonds [23].

### 2.3. Methylation Analysis

Methylation is an essential method to analyze linkages that form methoxyl groups on the free hydroxyl groups of polysaccharides [24]. After methylation of EPS-2, a series of methylated derivatives were identified based on a gas chromatography coupled with mass spectrometry (GC-MS) analysis. According to the retention time and the ion fragment characteristics in the GC-MS spectra, 1,2,5-tri-*O*-acetyl-3,4,6-tri-*O*-methyl-mannitol (residue A), 1,2,4,5-tri-*O*-acetyl-3,6-tri-*O*-methyl-mannitol (residue B), 1,5-di-*O*-acetyl-2,3,4,6-tetra-*O*-methyl-d-glucitol (residue C), 1,5,6-tri-*O*-acetyl-2,3, 4-tri-*O*-methyl-d-glucitol (residue D), 1, 5-di-*O*-acetyl-2, 3, 4, 6-tetra-*O*-methyl-galactitol (residue E), 1,4,5-tri-*O*-acetyl-2, 3-di-*O*-methyl-d-xylitol (residue F), and 2,3,4,5-tetra-*O*-acetyl-d-arabinitol (residue G) were detected (summarized in Table 1), indicating the presence of →2)–Manp-(1→, →2, 4)–Manp-(1→, Glc-(1→, →6)-Glcp-(1→, Gal-(1→, →4)–α-D-Xyl-(1→ and →2, 3, 5)–Ara-(1→, respectively. The molar ratio was 7.2:2.3:3.3:1.2:1.0:1.1:1.1, which was in agreement with the results of the GC analysis of monosaccharide components.

### 2.4. Nuclear Magnetic Resonance (NMR) Spectroscopy Analysis

**Figure 2:**
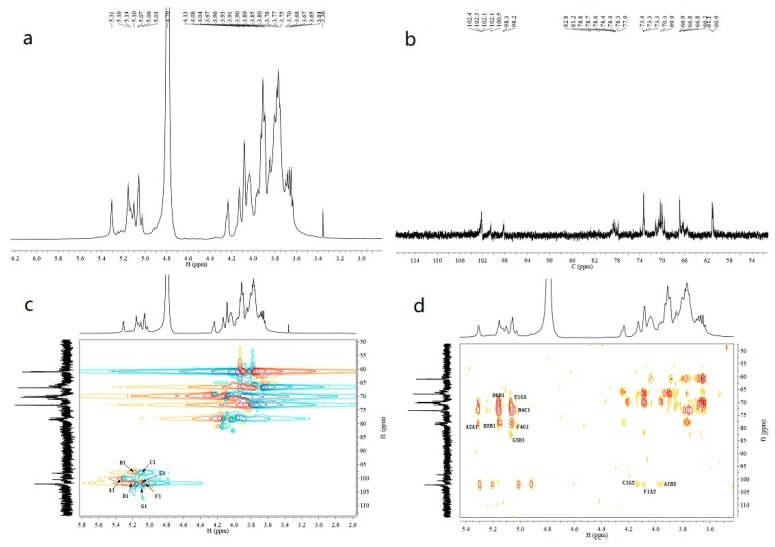

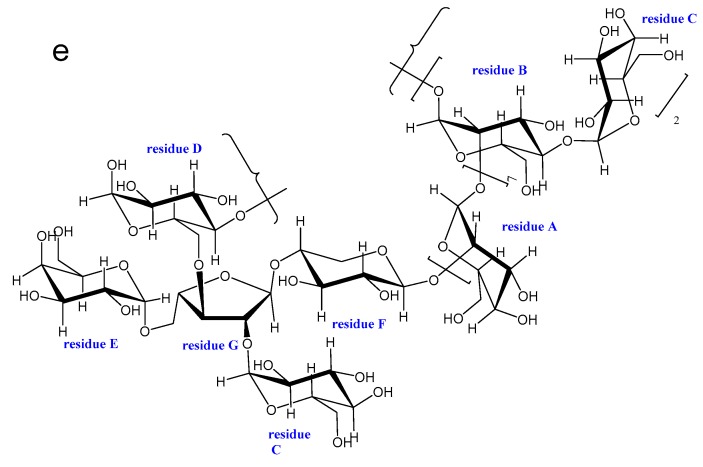
^1^H-NMR (**a**), ^13^C-NMR (**b**), HSQC (**c**), HMBC spectrum (**d**), and predicted repetitive structural unit (**e**) of EPS-2.

The structure of EPS-2 was further analyzed by NMR spectroscopy. The ^1^H, ^13^C, HSQC, and HMBC spectra of EPS-2 are shown in Figure 2. Several anomeric proton signals (5.31–5.03 ppm) appeared in the ^1^H-NMR spectrum. Other proton signals were located in the region of about 4.25–3.36 ppm, which were attributed to the protons of the C-2–C-6 of hexosyl glycosidic ring. The corresponding anomeric carbon signals (102.4–98.2 ppm) were identified in the ^13^C-NMR and HSQC spectra of EPS-2. These signals corresponded to seven types of residues (residue A–G, respectively), and this result was consistent with the GC-MS result.

The carbon and proton signals of residues A-G were grouped by comprehensive analysis, comparison of the NMR spectra, GC-MS data of EPS-2 and published literature [25,26,27,28]. The α/β configurations of residues were judged by the chemical shift and coupling constant of the anomeric proton [29,30]. In the HSQC spectrum, the anomeric signals at 100.5/5.30, 98.2/5.16, 98.3/5.07,102.4/5.10, 102.1/5.06, 102.0/5.03, and 102.3/5.07 ppm corresponded to residues A–G, respectively, which belonged to →2)–α-d-Manp-(1→, →2, 4)–α-d-Manp-(1→, α-d-Glcp-(1→, →6)-α-d-Glcp-(1→, α-Galp-(1→, →4)–α-d-Xylp-(1→, and →2, 3, 5)–β-d-Araf-(1→, respectively. Additionally, the adjacent carbon and hydrogen signals of each residue were assigned according to the HMBC spectrum. The data on carbon and hydrogen for EPS-2 are shown in Table 2.

Some points existed in HSQC and also showed in HMBC were removed, and the remained linkage sites between the residues were determined by analyzing the HMBC spectrum of EPS-2. In the HMBC spectrum, the peak at *δ* 78.5/5.30 ppm (AC2/AH1) suggested that there is a recurring C-2 linked O-1 of residue A structure in EPS-2. The signal at *δ* 78.3/5.16 ppm (B C2/B H1) has shown that C-2 is linked to O-1 of residue B, and the signal at *δ* 100.5/3.96 ppm (A C1/B H2) has indicated that C-2 of residue B is linked to O-1 of recurring residue A. Likewise, the linkages of residue F O-1 with residue A C-2, residue C O-1 with residue B C-4, and residue B O-1 with residue D C-6 were deduced by the signals at *δ* 102.0/4.11 ppm (FC1/AH2), 70.3/5.07 ppm (BC4/CH1), 66.2/5.16 ppm (DC6/BH1), respectively. In addition, signals at *δ δ*98.3/4.25 ppm (CC1/GH2), *δ* 82.8/5.10 ppm (GC3/DH1), *δ* 66.6/5.06 ppm (GC5/EH1), and *δ* 78.4/5.07 ppm (FC4/GH1) illustrate that O-1, C-2, C-3 and C-5 of residue G were linked to the C-4 of residue F, O-1 of residue C, O-1 of residue D, O-1 of residue E, respectively. The possible repetitive structure unit of EPS-2 was inferred and is shown in Figure 2e.

### 2.5. Effects of EPS-2 on the Viability of House Ear Institute-Organ of Corti 1 (HEI-OC1) Cells Treated with GM

**Figure 3:**
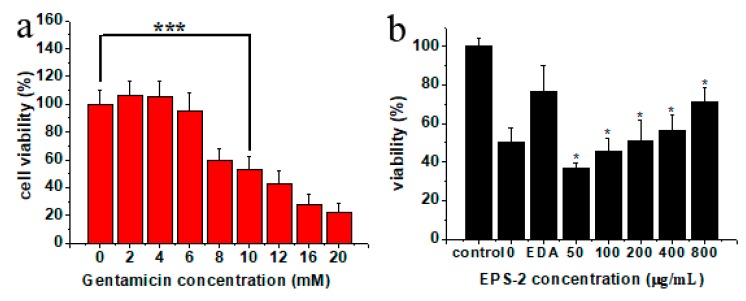
Effect of EPS-2 on GM-mediated decrease in viability of HEI-OC1 cells. (**a**) GM decreased cell viability dose dependently. GM (10 mM) treatment for 24 h decreased the cell viability by ca. 50%. (**b**) EPS-2 protected HEI-OC1 cells following GM (10 mM) treatment. * *p* < 0.05, *** *p* < 0.001 vs. control group. Results are shown as the mean ± SD (*n* = 6).

As evidenced by the MTT assay, the exposure to GM for 24 h decreased the cell viability in a dose-dependent manner. Cell viability was reduced by ca. 50% by 10 mM GM (Figure 3a). Thus, 10 mM GM was used subsequently. Cells were pretreated with 50, 100, 200, 400, and 800 μg/mL EPS-2 for 1 h before adding 10 mM GM. Control cells were treated with a vehicle (0.1% DMSO). Cell survival was determined after 24 h. In the control group, no cytotoxicity was observed at 0.1% DMSO. The exposure of HEI-OC1 cells to GM resulted in a significant reduction of cell viability, but cell pretreatment with EPS-2 significantly inhibited GM-mediated cytotoxicity in a dose-dependent manner, as shown in Figure 3b. In cells treated with GM only, EPS-2 at 50 μg/mL increased cell viability by 50% EDA (40 μM), a positive control also significantly increased cell survival.

### 2.6. Protective Effect of EPS-2 on Hair Cells in Neuromasts

Using the zebrafish lateral line as a model of hair cell death, we tested EPS-2 prevented GM-induced hair cell death. DASPEI was performed to stain mitochondria, and the mean number of hair cells in the four neuromasts (SO1, SO2, O1, and OC1) of the zebrafish larvae was calculated to quantitatively assess the changes. Aminoglycoside treatment caused nuclear fragmentation and reduced neuromast fluorescence, while protective compound could prevent fragmentation and preserve labeling intensity.

**Figure 4:**
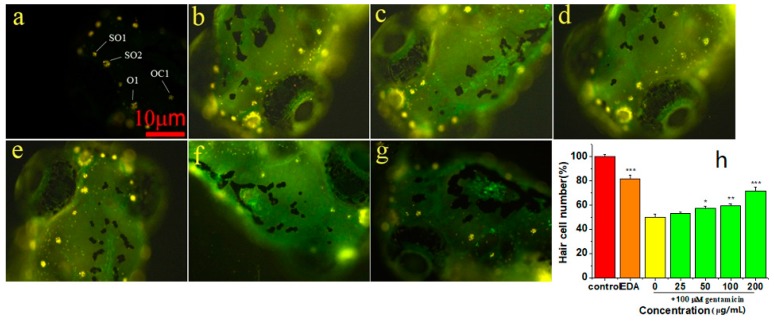
Analysis of hair cell damage by DASPEI assay (×10). (**a**) negative control: 36.62 ± 1.85 cells; (**b**) GM 100 μM treatment: 18.37 ± 2.26 cells; (**c**) EPS-2 25 μg/mL + GM 100 μM treatment: 19.37 ± 1.41cells; (**d**) EPS-2 50 μg/mL + GM 100 μM treatment: 21.00 ± 1.85 cells; (**e**) EPS-2 100 μg/mL + GM 100 μM treatment: 21.87 ± 1.55 cells; (**f**) EPS-2 200 μg/mL + GM 100 μM treatment: 26.25 ± 3.01 cells; (**g**) positive control: 30.00 ± 3.16 cells; and (**h**) EPS-2 protected hair cells following GM (100 μM) treatment. * *p* < 0.05, ** *p* < 0.01, *** *p* < 0.001 vs. control group). Results are shown as the mean ± SD (*n* = 8 fish per treatment). Scale bar = 10 μm.

From two independent screens, we found that EPS-2 protected hair cells from GM (Figure 4). We first determined whether 1 h of exposure to EPS-2 alone caused hair cell death, and the 400 μg/mL concentration of EPS-2 was revealed to have toxicity for hair cells compared to negative control, whereas 200 μg/mL EPS-2 had no toxicity for hair cells compared to the negative control group. Therefore, EPS-2 at a concentration of 200 μg/mL was expected to be applied as a maximal concentration in the following study. Pretreatment with 25, 50, 100, and 200 μg/mL EPS-2 resulted in significant protection of hair cells exposed to 100 μM GM compared to GM alone. The mean (± SD) number of hair cells in the neuromasts in the negative control group was 36.62 ± 1.85, and the viability of hair cells was set to 100%. In the model group (100 μM GM), the viability of hair cells was 50%; in the positive control group (0.5 μM EDA), the viability of hair cells was 82%; the protective effect of pre-treated EPS-2 increased in a dose-dependent manner until 200 μg/mL (the viability of hair cells was up to 72%) of concentration with a significantly large number of viable hair cells than that in the model group.

## 3. Experimental

### 3.1. Chemicals and Reagents

3-Aminobenzoic acid ethyl ester methanesulfonate (MS-222, CAS no. 886-86-2), 3-methy-1-pheny-2-pyrazoline-5-one (EDA, CAS no. 89-25-8), 2-(4-(dimethylamino)styryl)-*N*- ethylpyridinium iodide (DASPEI, CAS no. 3785-01-1) and 3-(4, 5-dimethylthiazol-2-yl)-2, 5-diphenyltetrazolium bromide (MTT, CAS no. 298-93-1) were obtained from Sigma-Aldrich (St Louis, MO, USA). Gentamycin (GM, CAS no.1405-41-0) was purchased from Dalilan Meilun Biotechnology Co., Ltd. (Dalian, China). The Hepes-buffered DMEM solution (pH 7.4, Gibco, 1×, sterile, CAS no. 21063-029) used in this study contained neither phenol red nor sodium pyruvate was purchased from Life Technologies Co. (Grand Island, NY, USA). High-glucose Dulbecco’s modified Eagle medium (DMEM) and Fetal bovine serum (FBS) were purchased from Thermo Fisher Scientific (Waltham, MA, USA). DEAE-cellulose 52 was purchased from Beijing Dingguo Changsheng Biotechnology Co., Ltd. (Beijing, China). Sephadex G-75 gel filtration medium was purchased from Shanghai Lanji Biotechnology Co., Ltd. (Shanghai, China). Trifluoroacetic acid (TFA), 1-phenyl-3-methyl-5-pyrazolone (PMP), standard monosaccharides (d-mannose, l-rhamnose, d-glucuronic acid, d-galacturonic acid, d-glucose, d-galactose, d-xylose, l-arabinose, and d-fucose), T-series dextrans of different molecular weights (T-5.2, T-11.6, T-23.8, T-48.6, T-148, T-273, T-410, T-668, and T-1400) and dialysis tubing (molecular weight cut off, 8000–14,000 Da) were obtained from Sigma-Aldrich (St. Louis, MO, USA). The reagents used in high-performance liquid chromatography (HPLC) and GC-MS were of chromatograph grade, and all other chemicals and reagents used were of analytical grade (AR).

### 3.2. Fungal Material Microbial Strain and Culture Conditions

The strain CSL-27 was isolated from the corm of saffron and identified as *Penicillium citreonigrum* by Beijing Dingguo Changsheng Biotechnology Co. Ltd. The strain was stored at China Center for Type Culture Collection (CCTCC) (Wuhan, China). The strain was activated on potato dextrose agar (PDA) slants, and then cultivated on a rotary shaker (TCYQ, Taicang Laboratory Equipment Factory, Jiangsu Province, China) constantly at 120 rpm and 28 °C for 14 days. The liquid culture medium contained 10 g/L glucose, 2 g/L peptone, 1 g/L yeast extract and 1 g/L NaCl with a pH of 6.5.

### 3.3. Cell Culture

HEI-OC1 cell line, derived from the organ of Corti was obtained from the House Ear Institute (Los Angeles, CA, USA). Cells were cultured in high-glucose DMEM, supplemented with 10% FBS at 33 °C and 10% CO_2_ in a humidified atmosphere without antibiotics. The cell incubator (HERAcell 150i) was derived from Thermo Fisher Scientific (Waltham, MA, USA).

### 3.4. Zebrafish Husbandry

Zebrafish (*Danio rerio*) embryos were produced by paired matings of AB wild-type adult fish from School of Pharmaceutical Sciences, Sun Yat-sen University and maintained in zebrafish facilities at School of Pharmacy, Guangdong Pharmaceutical University. Experiments were performed on 5–6 day old larval zebrafish maintained at 28 °C in a defined embryo medium (EM) containing 1 mM MgSO_4_, 120 μM KH_2_PO_4_, 74 μM Na_2_HPO_4_, 1 mM CaCl_2_, 500 μM KCl, 15 mM NaCl, and 500 μM NaHCO_3_ in distilled water at pH 7.2. This age range was selected due to the fact that hair cells in 5 day-old fish show mature responses to ototoxic insult, and the small fish size allows for high throughput screening of compounds in small volumes [31]. All procedures were approved by the appropriate Institutional Animal Care and Use Committee at Guangdong Pharmaceutical University.

### 3.5. Isolation and Purification of EPS-2

The supernatant from the culture of strain CSL-27 was collected and concentrated by a vacuum rotary evaporator (EYELA, Japan). The concentrated solution was treated with four volumes of cold 95% EtOH and kept overnight at 4 °C. The precipitate was separated and collected by centrifugation at 3000 rpm for 20 min and dissolved in distilled water and deproteinated by the Sevag method [32]. Finally, the precipitate was dialyzed in distilled water for 48 h at 4 °C and then freeze-dried to obtain a crude polysaccharide. The sugar content in the EPS was analyzed using phenol-sulfuric acid method with glucose as the standard [33].

The crude exopolysaccharide was purified using a column (2.6 × 95 cm) packed with Macroporous resin AB-8. Distilled water was employed as the mobile phase. The flow rate was 2 mL/min. Each fraction (10 mL) was collected and analyzed with the phenol-sulfuric acid reagent at 490 nm using a spectrophotometer [33]. The fractions, which coincided with the major peak, were collected together, concentrated at 60 °C with a rotary evaporator under vacuum, dialyzed (Mw cut off: 8000 Da) and lyophilized. The exopolysaccharide samples obtained by lyophilizing were dissolved in distilled water and fractionated on a pre-equilibrated DEAE-52 cellulose column (2.6 × 60 cm) equilibrated with distilled water and then eluted with aqueous NaCl solution (0.1 mol/L) at a flow rate of 1 mL/min. All the fractions were assayed for carbohydrate content by the phenol-sulfuric acid method and the fraction representing only one sharp peak was collected, dialyzed, concentrated and further purified using a Sephadex G-75 gel-filtration column (1.6 × 70 cm) by eluting with distilled water at a flow rate of 0.2 mL/min. Consequently, a fine exopolysaccharide, named EPS-2, was obtained. After freeze-drying, EPS-2 was available for use in the subsequent experiments.

### 3.6. Analysis of Physicochemical Characteristics

The molecular weight of EPS-2 was assessed by HPGPC on TSK-5000PWXL and TSK G-3000 PWXL gel columns (1.8 × 300 mm) in series (Tosoh Biosep, Tokyo, Japan). The columns were calibrated with dextran standards and a refractive index detector (Waters 2414, Milford, MA, USA), and eluted with 0.02 M KH_2_PO_4_ solution at a flow rate of 0.6 mL/min and column temperature of 35 °C. The molecular weight was estimated by reference to a calibration curve made by a set of standards dextran (*M*w: 1400, 668, 410, 273, 148, 48.6, 23.8, and 5.2 kDa) [34]. The sample was dissolved in 1 mL of distilled water and mixed with an equal volume of 4.0 M TFA. The sample was allowed to stand still for 4 h at 100 °C and the acid-hydrolyzed sample was filtered through a 0.45 μm syringe filter and the residual acid was removed through decompression and distillation with methanol for thrice [35]. The resulting monosaccharide compositions were determined by HPLC after precolumn derivatization with PMP using a Shimadzu HPLC system fitted with Phenomenex GEMINI-NX C_18_ HPLC column (4.6 nm × 250 mm) and Shimadzu prominence diode array detector. The sugar was identified by comparison with reference sugars (l-rhamnose, l-arabinose, d-fucose, d-xylose, d-mannose, d-galactose, d-glucose, d-glucuronic acid, and d-galacturonic acid). Calculation of the molar ratio of the monosaccharide was carried out on the basis of the peak area of the monosaccharide [24].

The morphology of EPS-2 was observed under a low vacuum scanning electron microscope (SEM, Philips Quanta-400, Netherlands). The dried exopolysaccharide powder was placed on a specimen holder with the help of double-sided adhesive tapes and then sputtered with the gold powder using a sputter coater. The sample was observed at magnifications of 800× and 1600× at an accelerating potential of 20 kV under low vacuum conditions.

### 3.7. FT-IR Analysis

FT-IR spectroscopy was used to determine the functional groups of the purified EPS. Infrared spectra of the purified EPS fraction were recorded in the 4000–400 cm^−1^ region using a FT-IR system (Perkin Elmer Spectrometer 100, Wellesley, MA, USA). The sample (10 mg) was mixed with 100 mg of dried potassium bromide (KBr) and compressed to prepare as a salt disc (10 mm diameter) for reading the spectrum further. The determinations were performed in two independent replicates and are reported as the mean with standard deviations.

### 3.8. Methylation Analysis

A methylation analysis was performed by the method of Hakomori with some modifications [15]. In brief, polysaccharide in dimethyl sulfoxide (DMSO) was methylated using NaH and iodomethane. After 6 h total hydrolysis with 2 M TFA at 105 °C, the methylated sugar residues were converted to partially methylated alditol acetates by reduction with NaBH_4_, followed by acetylation with acetic anhydride. The derived sugar residues were dissolved in 100 μL chloroform. Subsequently, partial methylated alditol acetates (PMAAs) were analyzed by GC-MS on the Shimadzu NTST system equipped with a TG WAXMS capillary column (30.0 m × 0.25 mm × 0.25 μm) (ThermoFinnigan, Silicon valley, CA, USA). The temperature was set to 50 °C, maintained for 3 min, then increased to 240 °C at a rate of 15 °C/min, and maintained at 240 °C for 20 min. Helium acted as the carrier gas, with the flow rate maintained at 1.0 mL/min. PMAAs were identified by the retention times and fragmentation patterns.

### 3.9. NMR Spectroscopy Analysis

^1^H-NMR and ^13^C-NMR spectra were recorded using a Bruker AVANCE IIIT600 NMR spectrometer at 25 °C. The sample (35 mg) was deuterium-exchanged by lyophilization two times with 99.97% D_2_O, and then was dissolved in 1.0 mL of 99.97% D_2_O. Acetone was taken as the internal standard (2.225 ppm for ^1^H and 31.07 ppm for ^13^C). ^1^H-NMR, ^13^C-NMR, ^1^H-^1^H COSY, HSQC, and HMBC were performed using the standard Agilent software.

### 3.10. Protection Assay

Hair cells act as sensory receptors for the auditory and vestibular systems in all vertebrates. Conventional vertebrate experimental animal models are well suited to detect and solve problems related to hair cell death and survival, but they are not suitable for drug screening. This is mainly because the inner ear is inaccessible and the alive time of inner ear cochlear tissue is very short in vitro [36]. As present, HEI-OC1 cell line is a mature and immortalized cell lines derived from the cochlea and vestibular tissue that has been shown to be sensitive to some known ototoxins, such as GM and cisplatin [37]. In addition, Hair cells in the inner ear of mammals are similarity with the pathway activated by ototoxicity exposure in zebrafish lateral line, but hair cells in zebrafish regenerate following ototoxicity exposure unlike mammals. Therefore, the zebrafish lateral line is an excellent model for drug screening that modulates hair cell survival, an intractable approach in mammalian systems [3]. In this experiment, HEI-OC1 cell line and zebrafish model were effectively combined for the activity assay of EPS-2.

#### 3.10.1. Cell Viability Assay

Cell viability was measured using the MTT assay as described previously [38]. HEI-OC1 cells were seeded at a density of 1 × 10^4^ cells/well in a 96-well plate and cultured overnight. To investigate the effect of EPS-2 on cell viability, HEI-OC1 cells were treated with 50, 100, 200, 400, and 800 μg/mL EPS-2 for 1 h, before being exposed to GM. When the cells were confluent, the culture medium was replaced with medium containing GM, which was the calculated half-maximal inhibitory concentration (IC_50_). After one day of incubation, 100 µL MTT was added to each culture well and the 96-well plate was incubated at 33 °C in an atmosphere of 10% CO_2_ for 4 h. EDA (40 μM) was used as a positive control. The effect of EPS-2 on viability at each concentration was calculated as a percentage of the control activity from the absorbance values. Absorbance at 490 nm was measured using a Microplate spectrophotometer (RT-2100C, Shenzhen Rayto Life Science Co., Ltd., China) for cell viability and the average OD in control cells was taken as 100% of viability. A final concentration of 10 mM GM was selected to damage the HEI-OC1 cells in the following experiments.

Cell relative viability (%) = OD_experiment_/OD_control_ × 100% (OD_blank_ was used to zero)

Six wells were used for each EPS-2 concentration and three independent experiments were performed.

#### 3.10.2. Assay of Zebrafish Neuromast Hair Cell Protection

At 5–6 days post-fertilization (dpf) AB zebrafish larvae were raised at 28.5 °C in Petri dishes and transferred to cell culture baskets placed in 96-well culture plates in groups of 3–4 fish per basket. The larvae were exposed to EPS-2 at the following concentrations: 25, 50, 100, 200, and 400 μg/mL for 1 h for the experimental group. A negative control group with no additional sample was also established. The larvae were then washed with the EM three times and anesthetized using 40 μg/mL MS-222 for 5 min as described in previous publications [39]. The mean count of hair cells was calculated within four neuromasts (SO1, SO2, O1, and OC1) on one side of each fish at a 10× magnification using a Zeiss inverted fluorescence microscope (Carl ZEISS AG, Germany) for each group (*n* = 8). All zebrafish were alive and no abnormal developments were observed.

Then the 5–6 dpf zebrafish larvae were pretreated with EPS-2 for 1 h at concentrations of 25, 50, 100, and 200 μg/mL followed by treatment with 100 μM GM, respectively. Following 1 h GM exposure, the larvae were rinsed briefly with EM, and incubated in a staining agent (0.005% DASPEI) for 15min, rinsed three times with fresh EM and anesthetized in 40 μg/mL MS-222 for 5 min. The hair cells within the above-mentioned four neuromasts were examined. Each neuromast was scored for presence of a normal compliment of hair cells, with reduced or absent DASPEI staining indicating a reduction in the number of hair cells. Composite scores were calculated for the larvae in each treatment group, normalized to the control group and expressed as % hair cell survival. Negative controls were treated with GM while positive controls were treated with 0.5 μM EDA.

#### 3.10.3. Statistical Analysis

All data were presented as the mean ± standard deviation. One-way analysis of variance (ANOVA) was used for multiple comparisons; *p* < 0.05 was considered statistically significant. Statistical analysis was performed with IBM SPSS 21.0 for Windows (IBM, Armonk, NY, USA).

## 4. Conclusions

Hearing loss is the one of the most common sensory disorders in humans, and a large number of cases are due to hair cell damage caused by ototoxicity drugs such as GM. Therefore, it is great significance to identify agents and their mechanisms that protect hair cells from ototoxicity damage [40]. Saffron has strong biological activities, and the active part is concentrated in the stigma, and the amount is too small to be detrimental to further research. Thus, endophytic fungus is a good substitute for studying saffron. Moreover, many literatures have reported that endophytic exopolysaccharides have unique charms and effective activities. We previously reported the antioxidant activity of crude exopolysaccharide extracted from fermentation mycelia of saffron. The characterization of polysaccharides of the endophytic fungus had great significance for the further structure-function relationship study, and the development and application of the endophytic polysaccharide. Therefore, we purified the polysaccharide with DEAE-52 cellulose and Sephadex G-75 columns, and a new water-soluble endophytic polysaccharide EPS-2 with a molecular weight of 40.4 kDa was obtained. The results of monosaccharide composition, FT-IR spectroscopy, GC-MS and NMR analyses suggested that EPS-2 is composed of →2)–Manp-(1→, →2, 4)–Manp-(1→, Glc-(1→, →6)- Glcp-(1→, Gal-(1→, →4)–α-d-Xyl-(1→, and →2, 3, 5)–Ara-(1→. The possible repetitive structural unit of EPS-2 was inferred. The most effective concentration of EPS-2 for attenuating GM-induced HEI-OC1 cell damage was 200 μg/mL (50% cell viability), and EPS-2 protected hair cells from a concentration of 25 μg/mL (50% hair cell number) in a zebrafish model. In conclusion, the study reports the systematic purification, structural identification, and the in-vitro testing of its protective effects on hair cells against GM toxicity of EPS-2 and notes its potential as a natural candidate lead for new drugs to combat hearing loss that can be utilized in the pharmaceutical and healthcare industries.

## Figures and Tables

**Table 1:** Glycosidic linkage composition of methylated EPS-2.

PMAA	Mass Fragments (*m*/*z*)	Molar Ratio	Linkage Type
3,4,6-Me_3_-Manp ^a^	87, 101, 117, 129, 161, 189, 233	7.2	→2)-d-Manp-(1→
3,6-Me_2_-Manp	43, 71, 87, 129, 173, 189, 233	2.3	→2, 4)-d-Manp-(1→
2,3,4,6-Me_4_-Glcp	45, 71, 87, 101, 117, 129, 145, 161, 205	3.3	d-Glcp-(1→
2,3,4-Me_3_-Glcp	58, 71, 87, 99, 101, 117, 129, 161, 189, 233	1.2	→6)-d-Glcp-(1→
2,3,4,6-Me_4_-Galp	87, 101, 117, 129, 189, 233	1.0	d-Galp-(1→
2,3-Me_2_-Xylp	43, 87, 101, 117, 129, 189	1.1	→4)-d-Xylp-(1→
Araf	73, 85, 115, 145, 158, 187, 217	1.1	→2, 3, 5)-d-Araf-(1→

^a^ 3, 4, 6-Me_3_-Manp = 1, 2, 5-Tri-*O*-acetyl-3,4,6-tri-*O*-methyl-mannitol.

**Table 2:** Assignment of ^13^C-NMR and^1^H-NMR chemical shifts of EPS-2.

No.	Glycosyl Residues	Chemical Shifts, δ (ppm)					
		C1/H1	C2/H2	C3/H3	C4/H4	C5/H5	C6/H6
A	→2)–α-d-Manp-(1→	100.5 5.30	78.5 4.11	70.6 3.97	66.8 3.65	73.9 3.80	63.4 3.91
B	→2, 4)–α-d-Manp-(1→	98.2 5.16	78.3 3.96	70.1 4.07	70.3 3.89	73.6 3.76	66.9 3.85, 3.77
C	α-d-Glcp-(1→	98.3 5.07	70.4 3.80	73.3 3.85	76.7 4.08	70.1 3.90	61.0 3.81, 3.75
D	→6)-α-d-Glcp-(1→	102.4 5.10	73.3 3.36	73.5 3.60	70.4 3.62	76.8 4.08	66.2 3.75
E	α-d-Galp-(1→	102.1 5.06	70.2 3.82	73.2 4.13	71.2 4.02	73.3 4.04	61.1 3.72
F	→4)–α-d-Xylp-(1→	102.0 5.03	75.3 3.82	76.3 3.95	78.4 3.73	65.5 3.65	
G	→2, 3, 5)–β-d-Araf-(1→	102.3 5.07	81.1 4.25	82.8 4.02	81.1 4.23	66.6 3.96, 3.88	
